# Automated Network Analysis Identifies Core Pathways in Glioblastoma

**DOI:** 10.1371/journal.pone.0008918

**Published:** 2010-02-12

**Authors:** Ethan Cerami, Emek Demir, Nikolaus Schultz, Barry S. Taylor, Chris Sander

**Affiliations:** 1 Computational Biology Center, Memorial Sloan-Kettering Cancer Center, New York, New York, United States of America; 2 Tri-Institutional Training Program in Computational Biology and Medicine, New York, New York, United States of America; University of Pennsylvania, United States of America

## Abstract

**Background:**

Glioblastoma multiforme (GBM) is the most common and aggressive type of brain tumor in humans and the first cancer with comprehensive genomic profiles mapped by The Cancer Genome Atlas (TCGA) project. A central challenge in large-scale genome projects, such as the TCGA GBM project, is the ability to distinguish cancer-causing “driver” mutations from passively selected “passenger” mutations.

**Principal Findings:**

In contrast to a purely frequency based approach to identifying driver mutations in cancer, we propose an automated network-based approach for identifying candidate oncogenic processes and driver genes. The approach is based on the hypothesis that cellular networks contain functional modules, and that tumors target specific modules critical to their growth. Key elements in the approach include combined analysis of sequence mutations and DNA copy number alterations; use of a unified molecular interaction network consisting of both protein-protein interactions and signaling pathways; and identification and statistical assessment of network modules, i.e. cohesive groups of genes of interest with a higher density of interactions within groups than between groups.

**Conclusions:**

We confirm and extend the observation that GBM alterations tend to occur within specific functional modules, in spite of considerable patient-to-patient variation, and that two of the largest modules involve signaling via p53, Rb, PI3K and receptor protein kinases. We also identify new candidate drivers in GBM, including AGAP2/CENTG1, a putative oncogene and an activator of the PI3K pathway; and, three additional significantly altered modules, including one involved in microtubule organization. To facilitate the application of our network-based approach to additional cancer types, we make the method freely available as part of a software tool called NetBox.

## Introduction

Glioblastoma multiforme (GBM) is the most common and aggressive brain tumor in humans [1]–[3], and the first cancer type to undergo comprehensive genomic characterization by The Cancer Genome Atlas (TCGA) project [4]. Glioblastoma is classified into two broad categories: primary and secondary. Primary glioblastomas (accounting for 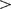
90% of cases and most of the TCGA cases profiled) manifest *de novo* without prior evidence of preexisting tumor; secondary glioblastomas develop through malignant progression from lower grade astrocytomas [3]. Prognosis for glioblastoma patients remains dismal, as most patients die within one year after diagnosis [3] and generally respond poorly to current therapeutic approaches [4], [5].

High-throughput cancer genomic studies, such as those being organized by the TCGA and the International Cancer Genome Consortium (ICGC) [6], are now enabling the research community to examine the cancer genome in a comprehensive and unbiased manner [7]. These efforts will soon lead to a comprehensive catalog of altered genes, altered biological processes and, by implication, therapeutic vulnerabilities in cancer. For example, the TCGA GBM project has cataloged somatic mutations and recurrent copy number alterations in GBM, and has identified frequent alterations in the p53, RB, PI3-kinase (PI3K) and receptor tyrosine kinase (RTK) signaling pathways [4].

A fundamental and open challenge in cancer genomics is the ability to distinguish “driver” from incidental “passenger” mutations. To first approximation, driver mutations are those that confer the tumor with some selective advantage in growth and contribute to tumorigenesis, whereas passenger mutations do not [8]. A number of approaches have been developed to distinguish drivers from passengers, including those that examine the rate of synonymous versus non-synonymous mutations [8], those that predict the functional consequence of mutations [9], and newer approaches that assess the overall rate of recurrence, based on combined rates of sequence mutation and copy number alteration [10]. A more recent approach by Torkamani et. al. [11] sought to identify cancer drivers by identifying an enrichment of rare cancer mutations within network modules reconstructed from gene expression studies. Here, we also present a network-based approach to identifying driver mutations in cancer, apply this approach to GBM, and discuss potential applicability to other cancer types.

Our network-based approach is based on the hypothesis that cellular networks are modular, and consist of inter-connected proteins responsible for specific cellular functions [12], [13]. It is further based on the hypothesis that the cancer phenotype is based on the inability of multiple genetic functional modules to carry out their basic functions, and that functional modules are critical to the “hallmarks of cancer”, including self-sufficiency in growth signals, evasion of apoptosis, sustained angiogenesis, tissue invasion and metastasis [14]. Furthermore, different combinations of perturbed genes can incapacitate each module [15], and each tumor can perturb individual genes via multiple mechanisms including sequence mutations, copy number alterations, gene fusion events, or epigenetic changes. Evidence for such universality at the module-level, but diversity at the genetic level can be seen in multiple cancer types, including in glioblastoma. For example, nearly all GBM tumors contain alterations in the p53 tumor suppressor pathway, but individual tumors exhibit diverse mechanisms for pathway alteration – mutation or homozygous deletion of *TP53*, mutation or homozygous deletion of *CDKN2A*/ARF, or amplification of *MDM2*/*MDM4*. If tumors frequently target biological modules that execute key biological processes, and network knowledge about such modules is available, we hypothesized that it would be possible to algorithmically identify frequently perturbed modules, and from these modules identify candidate driver mutations.

## Results and Discussion

### A Network-Based Approach for Distinguishing Driver from Passenger Mutations

**Figure 1:**
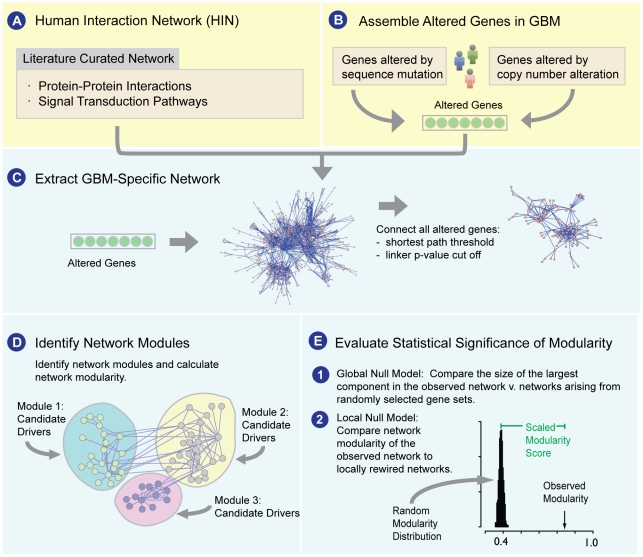
Overview of the network approach for identifying oncogenic processes and candidate driver genes in GBM. We began by creating a literature curated Human Interaction Network (HIN) derived from protein-protein interactions and signaling pathways (A), and assembling genomic alterations in GBM (B). We then extracted a GBM-specific network of altered genes (C), which was then partitioned into network modules (D). We assessed the level of connectivity seen within the GBM network by using (E1) a global null model to compare the size of the largest component in the observed network v. networks arising from randomly selected gene sets; and (E2) a local null model to compare network modularity of the observed network to locally rewired networks.

We performed integrated network analysis to identify frequently altered network modules and candidate driver mutations in glioblastoma. The network analysis is summarized in Figure 1. We began by constructing a global Human Interaction Network (HIN). Due to the potentially high rate of false positives and false negatives associated with high-throughput protein-protein interaction detection techniques [16] and natural language processing (NLP) algorithms [17], we chose to construct the HIN of literature curated data sources only (see Methods). To cover increased network territory, we also chose to create a unified HIN consisting of both protein-protein interactions and signaling pathways. The final network consists of genes, represented as nodes, and interactions, represented as edges. Interactions represent any functional association between two genes, such as a direct protein-protein interaction, membership in the same complex, or a state change event, such as a phosphorylation event. Redundant edges in this network may exist if multiple data sources have evidence for the same interaction. Since the module detection algorithm described below does not take into account multiple lines of evidence or self-directed edges, all redundant edges were collapsed into single edges, and all self-directed edges were pruned from the network.

To gain a global view of genomic alterations occurring in glioblastoma, we restricted our analysis to the 91 TCGA GBM cases, for which both copy number and sequence mutation data were available. Seven of these cases were treated with adjuvant chemotherapy and classified as hypermutators, and were excluded from the analysis. To focus on minimally recurrent alterations, we also only included genes that were altered in two or more of the final 84 cases. As was the case in the original TCGA pathway analysis, only unequivocal genetic alterations were included [4]. This includes validated non-synonymous somatic nucleotide substitutions, and likely homozygous deletions and multi-copy amplifications, as determined by the RAE algorithm (see Methods).

A total of 517 genes passed the frequency threshold. Of these, 274 genes had interactions in the human interaction network (HIN). We next tried to connect these 274 genes into a network, based on prior known interactions in the HIN. Our goal was to include interactions between altered genes, and to simultaneously identify biologically informative “linker” genes which were not in the original altered list, but which were statistically enriched for connections to member of the GBM gene list. By including such linkers, we aimed to connect more genes in the original gene list, and to provide greater biological context for module discovery and interpretation. For each pair of genes, we found all shortest paths of lengths 1 and 2 connecting the two genes in the HIN. If two genes are connected via a shortest path of 1, they are directly connected via an interaction. If two genes are connected via a shortest path of 2, there may be multiple paths of length two, each of which includes a unique linker gene. To retain information rich linker genes only, we applied a statistical threshold test for local enrichment. The statistical threshold uses the global degree of each linker gene within the HIN and the hypergeometric distribution to assess the probability that the linker gene would connect to the observed number of altered genes by chance alone. After FDR correction, linker genes which do not pass a p-value threshold of 0.05 were pruned from the network. Using a shortest path threshold of 2 and a FDR-adjusted p-value cut-off of: 0.05, we were able to connect 66 GBM altered genes and identify 6 linker genes.

To assess the level of global connectivity in the observed GBM network, we compared the size (number of nodes and edges) of the largest component in the network to the largest component generated by randomly selected sets of genes known to be present in the HIN. At each of 1000 iterations, we randomly selected 274 genes from the HIN and connected them via the same shortest path threshold and p-value cut-off parameters. This test showed that the GBM network is highly connected, more so than expected by chance (number of nodes  = 55, p-value: 0.014; number of edges  = 135, p-value: 0.01).

Finally, we partitioned the GBM network into network modules or communities — clusters of network nodes joined together in tightly knit groups, between which there are only looser connections [18]. This community structure or network modularity has been identified in diverse network systems, including social [18], [19], scientific collaboration [20], metabolic [21], and molecular interaction networks [22]–[24]. Numerous network module algorithms have been proposed, including those that take into account network topology alone, and those that take into account network topology plus additional biological information, such as correlated gene expression or subcellular localization [23], [25]–[27]. We chose to use the widely used edge betweenness algorithm by Girvan and Newman [18] for modularity detection. In this final step, we also calculated the modularity of the partitioned GBM network. This is a well-defined measure in network analysis that quantifies the extent of modularity seen in an observed network (see Methods).

Using the Newman-Girvan module detection algorithm, we detected a total of 10 modules, with an overall network modularity of 0.519. Random graphs and scale-free graphs can exhibit a relatively high degree of modularity [28]. We therefore chose to assess the statistical significance of the observed network modularity in relation to a null model of random networks of the same size and same degree distribution. To do so, we performed 1000 random simulations — in each simulation, the observed GBM network was locally rewired, such that all genes had exactly the same number of connections as before, but the choice of interaction partners was random [29]. For each random network, the network modularity score was recalculated, and the observed modularity score was then converted to a z-score or a scaled modularity score [30]. One thousand local rewiring simulations found an average network modularity of 0.296, with a standard deviation of 0.058. This resulted in a scaled modularity score of: 3.84, providing evidence that the GBM network is more modular than random.

### Properties of the GBM Network

**Figure 2:**
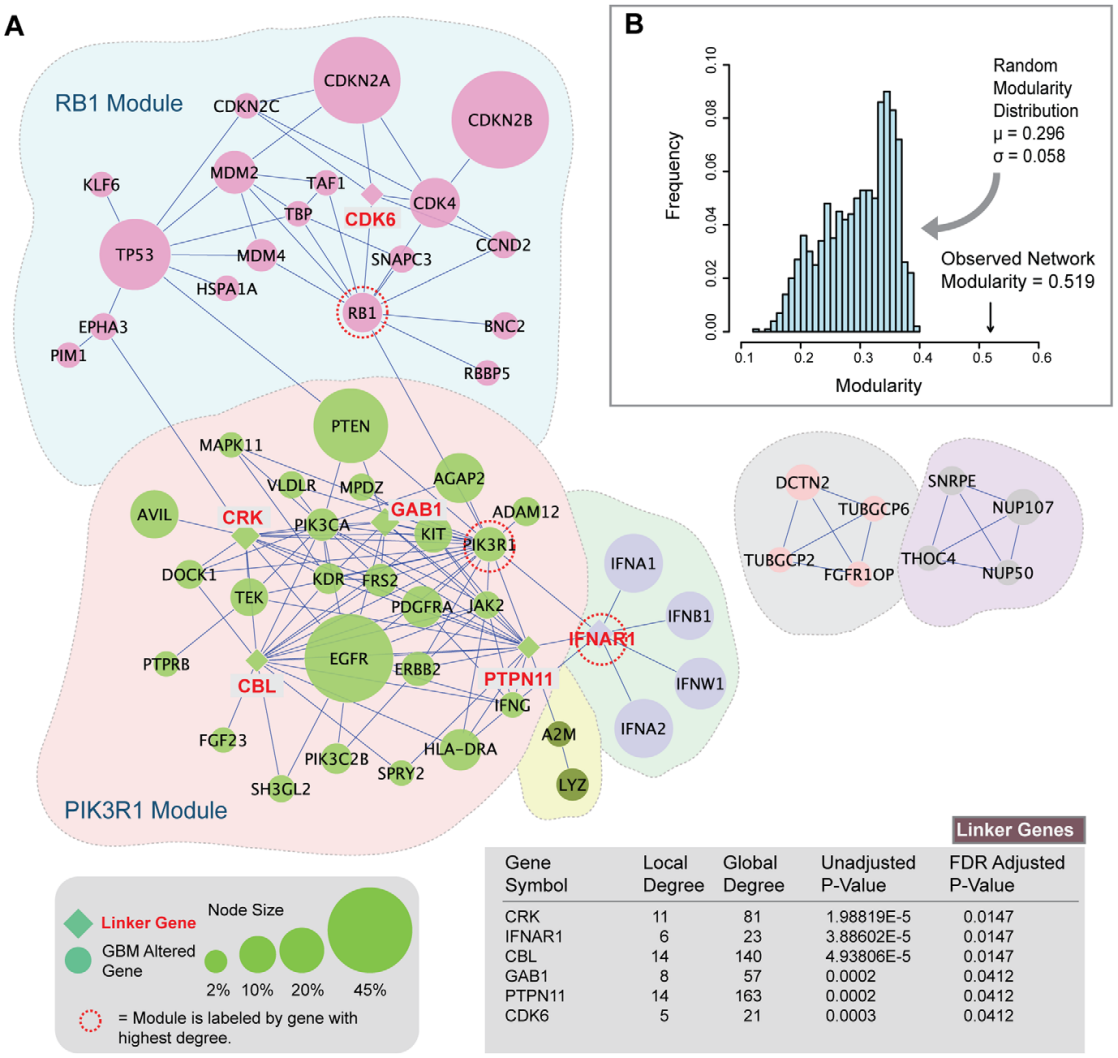
Network modules identified in GBM. (**A**) Modules are densely connected sets of altered genes that may reflect oncogenic processes. A total of 10 modules were identified, the largest of which are shown. Linker genes, indicated in red, are not altered in GBM, but are statistically enriched for connections to GBM-altered genes. (**B**) The observed modularity of the GBM network (0.519) is compared with 1000 randomly rewired networks (average 0.296, standard deviation 0.058). This results in a z-score, or scaled modularity score, of 3.84.

The main set of network modules identified in glioblastoma is shown in Figure 2. From these modules, we can make two initial observations. First, the network-based approach identifies many of the same driver candidates as the original TCGA frequency-based approach used to assess mutational significance. The original TCGA approach (with false discovery rate 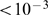) initially identified eight genes as significantly mutated. Notably, seven of these genes appear within the GBM network, and all seven appear within the two largest modules (NF1 does not appear within the network). Additional, while no strict statistical cut-off was used to assess genes individually altered by copy number alteration in the original analysis, many of the genes that are targets of high-level focal amplification or homozygous deletions, including *EGFR*, *CDK4*, *CDKN2A/B*, *PTEN*, *MDM2* and *MDM4* are all also identified within the GBM network.

**Figure 3:**
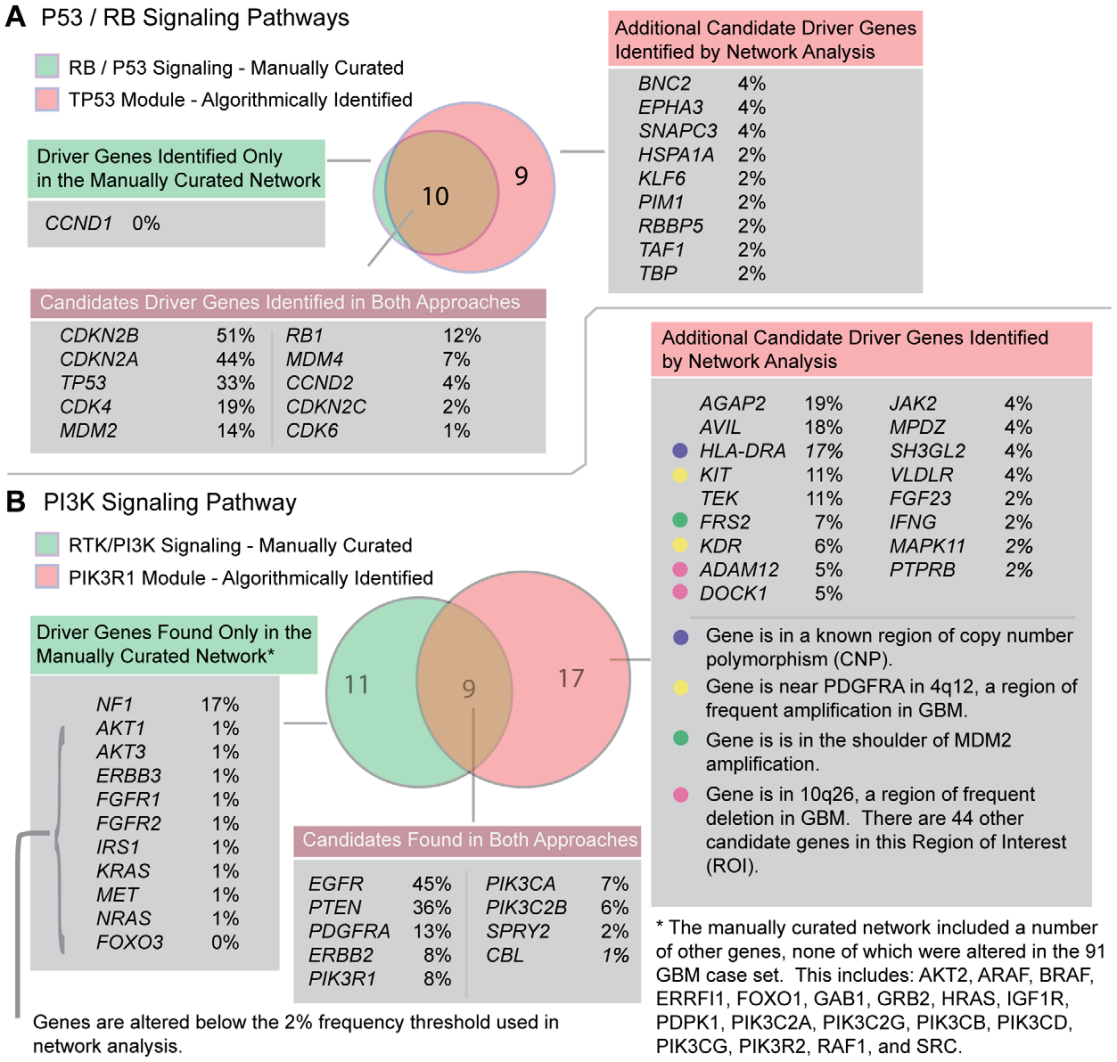
Automated network analysis approach is in close agreement with previous manually curated pathway analysis approach. The original pathway analysis of TCGA glioblastoma datasets was derived by mapping observed gene alterations onto a manually curated GBM-specific network, based on the glioblastoma literature. This non-algorithmic analysis identified driver alterations in the p53, RB and PI3K pathways. Our automated network analysis approach is in close agreement with these results (top: P53/Rb; bottom: PI3K). The one main exception is that network analysis does not identify NF1 as a participant in the PI3K module. Additional candidate driver genes identified by network analysis, including AGAP2, are identified and annotated on the right. Percentage values after each newly identified candidate driver indicate percent of cases with genetic alterations (sequence mutations, homozygous deletions, or multi-copy amplifications) across the 84 TCGA GBM cases analyzed.

Second, the two largest modules identified by network analysis correspond very closely to the three critical signaling pathways identified in the original TCGA pathway analysis. These modules also correspond well to genetic and pathway alterations known to GBM biology prior to TCGA analysis [2]. For example, the p53 tumor suppressor pathway, which prevents the propagation of unstable genomes, is frequently altered in glioblastoma [2]. Alterations within the p53 pathway include mutations and deletions of *TP53*, homozygous deletion of *CDKN2A*, and amplifications of *MDM2* and *MDM4* [2], [4]. The algorithmically identified RB1 module contains all of these known genetic alterations (Figure 3A). Glioblastomas also nearly universally circumvent cell cycle inhibition through genetic alterations to the RB pathway [2]. These alterations include mutations in *RB1* [31], [32], amplifications of *CDK4* [33], *CDK6* [34] *CCND1* or *CCND2*; and homozygous deletions of *CDKN2A*, *CDKN2B*, or *CDKN2C* [4]. All of these alterations, with the exception of *CCND1*, which did not meet our frequency threshold, are also clustered together within the RB1 module identified by network analysis (Figure 3A). Notably, *CDK6* was not included in the original input list, but was identified as a statistically significant linker gene.

The largest and most densely inter-connected module identified is the PIK3R1 module. This module contains many members of the phosphatidylinositol 3-Kinase-AKT pathway, also a frequent target of disruption in glioblastoma [2]. Major downstream effects of PI3K/AKT activation include cell growth, proliferation, survival and motility, all factors that drive tumor progression. Known alterations in the pathway include frequent alterations to receptor tyrosine kinases, homozygous deletion of *PTEN*, homozygous deletion of *NF1*, mutations in *AKT*, and alteration to components of the PI3K complex [2], [4]. The PIK3R1 module includes all of these alterations, with the exception of *NF1* and *AKT*. Identified members include *PTEN*, receptor tyrosine kinases: *EGFR*, *PDGFRA*, and *ERBB2*. It also includes two subunits of the Class I PI3K complex, including the p110 catalytic subunit *PIK3CA*, and the p85 regulatory subunit *PIK3R1* (Figure 3B).

To test the robustness of these initial observations, we performed network analysis of mutated genes discovered in a separate unbiased re-sequencing effort in GBM [35]. For this, we used the set of 40 GBM candidate cancer genes (CAN-genes) that are most likely to be cancer drivers. Using a shortest path threshold of two and a linker p-value cut off of 0.001, we were able to place 10 CAN genes into a network context and identify 9 significantly enriched linker genes. Network partitioning was then able to identify two main modules, one corresponding to the components of the PI3K pathway (including *EGFR*, *PTEN*, *PIK3CA*, and *PIK3R1*), and one corresponding to the components of the TP53/RB pathway (including *TP53*, *CDKN2A*, *CDK4* and *RB1*) (data not shown). Despite the much smaller sample size used in the Parsons study, network analysis was also able to identify 6 of the 8 genes identified as significantly mutated in the TCGA study, and the main two-module signature is also present in the Parsons data set. From this, we can conclude that the the module signature is robust across both data sets and the modules are frequently altered across patients profiled in both studies.

In summary, a network-based approach identifies many of the same candidates as the original frequency-based approach used to assess mutational significance. Furthermore, GBM alterations cluster within non-random modules, and the modules are robust across different studies; and a network-based approach can automatically identify and extract biologically relevant GBM modules, which correspond closely to prior known GBM biology.

### Identification of *AGAP2* and Additional Candidate Drivers in the PIK3R1 Module

As has been noted by others, clustering of networks into modules can be used to direct experimental research by providing a functional context for previously uncharacterized genes [23]. In our case, network module analysis identifies new candidate driver genes within previously identified p53/RB and PI3K signaling modules, and within newly identified modules in glioblastoma. For example, within the PIK3R1 module, the most notable new driver candidate is *AGAP2*, a gene which has been previously implicated in human glioblastoma [36], but was not reported in the first TCGA pathway analysis [4]. *AGAP2*, also known as *PIKE* and *CENTG1*, is amplified in 19% of the GBM cases, and is is frequently co-amplified with Cyclin-dependent kinase 4 (CDK4) in a variety of human cancers, including sarcoma, glioblastoma, and neuroblastoma [37]. As CDK4 is known to phosphorylate Rb, and is a known oncogene, the independent oncogenic role of *AGAP2* is in question. However, recent evidence indicates that PIKE-A, one of the three isoforms of *AGAP2*, specifically binds to active AKT [37], and that PIKE-A is a proto-oncogene capable of promoting cell proliferation and invasion [38]. AGAP2 may therefore indeed be a driver in GBM oncogenesis, and may represent an alternative or additional means by which glioblastoma tumor cells activate the PI3K pathway, and its downstream effects, including cell proliferation, inhibition of apoptosis, and tumor invasiveness. However, as with all such predictions regarding driver genes, definitive evidence can only be provided by further functional studies.

Other new candidate driver genes altered in at least 5% of GBM cases, but not detected by a previous frequency-based mutation significance test or previous pathway analysis include: *AVIL*, *KIT*, *TEK*, *FRS2*, and *KDR*. *AVIL* is implicated primarily by multi-copy amplifications (18% of cases), is in the shoulder of the *CDK4*/*AGAP2* amplicon, and may play a role in the development of neuronal cells that form ganglia [39]. *KIT* and *KDR* are both located in the shoulder of the *PDGFRA* amplicon, which may represent the true target of amplification. However, these genes may represent additional drivers and targets of amplification. For example, *KIT* has been identified as a proto-oncogene, and has been previously implicated in glioblastoma [40]–[42]. *KDR* encodes one of the two receptors of the Vascular Endothelial Growth Factor (VEGF), and maintains a key role in regulating angiogenesis-related functions [43]. Notably, the *TEK* receptor tyrosine kinase (located in the shoulder of the CDKN2A deletion), is also involved in angiogenesis, and is the receptor for angiopoietin-1 [44]. As is the case with AGAP2, and all other predictions regarding driver genes, definitive evidence regarding the role of these genes in GBM oncogenesis can only be provided by further functional studies.

### Identification of Additional Modules and Candidate Drivers

**Figure 4:**
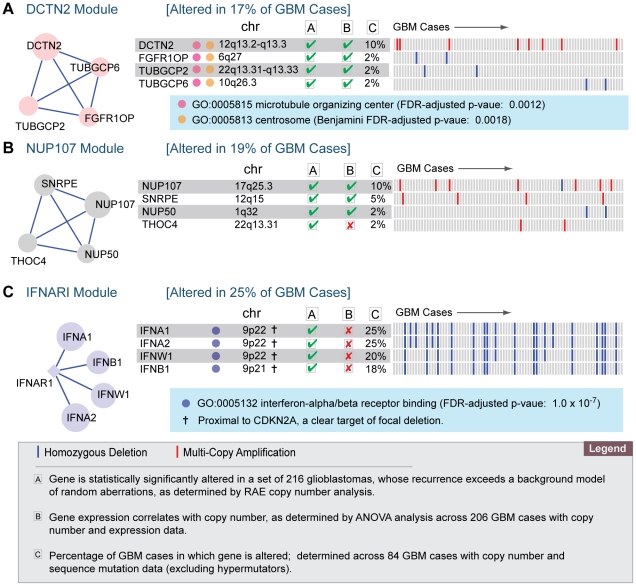
Network analysis identifies three additional altered modules, including the DCTN2 module, which is involved in microtubule organization. Each of the altered modules is implicated by homozygous deletions or multi-copy amplifications across the 84 analyzed GBM cases. Each module is annotated with Gene Ontology enrichment, chromosome location, statistical significance of copy number alteration against a background model of random aberrations, as determined by RAE copy number analysis; assessment of correlation between copy number and mRNA expression, and genomic signature across 84 GBM cases.

In addition to the two main modules, network analysis also identified eight additional modules. Three of these modules contained four or more genes, and are briefly summarized below and in Figure 4.

The DCTN2 module is altered in 17% of all GBM cases, and includes four genes: *DCTN2*, *TUBGCP2*, *TUBGCP6*, and *FGFR1OP*. All four of the genes are located in the centrosome and microtubule organizing center (FDR adjusted p-value: 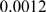). Centrosomes are critical to organizing the interphase cytoskeleton and the bipolar mitotic spindle, both of which are critical to ensuring the correct segregation of chromosomes during cell division. Centrosomes also serve as a “command center” for integrating multiple cell cycle and repair signals, including CHEK1, CHEK2, ATM, BRCA1, and ATR in response to DNA damage [45]. We hypothesize that alteration of centrosome-related genes cause dysregulation of chromosomal segregation and/or normal DNA damage response, either of which could lead to increased genomic instability in GBM.

The NUP107 module is altered in 19% of all GBM cases, and includes four genes: *SNRPE*, *THOC4*, *NUP107*, *NUP50*. The module is not enriched for any GO processes, but two of the genes, *NUP50* and *NUP107* are components of the nuclear pore complex. The IFNAR1 module is altered in 25% of all GBM cases, and includes four altered genes: *IFNA1*, *IFNA2*, *IFNB1*, and *IFNW1* and one linker gene: *IFNAR1*. Notably, all four of the genes are involved in interferon-alpha/beta receptor binding (FDR adjusted p-value: 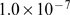), but all genes are also located on chromosome 9p, proximal to *CDKN2A*, which is a clear target of focal deletion. The genes could therefore be passenger deletions and deleted as a consequence of their proximity to CDKN2A.

### Availability of Software

To facilitate the application of our network-based approach to additional cancer types, we have made our software freely available as part of a command line tool, called NetBox. NetBox is pre-loaded with the Human Interaction Network (HIN) defined above, and provides a simple command line interface for connecting genes into a network, identifying linker genes, partitioning the network into modules, and executing the random background models. Results are then made available to the end user as an HTML web page and a series of network and attribute files, which can be loaded into Cytoscape [46] for visualization and further analysis. The NetBox software, user guide, and GBM data sets are available for download at: http://cbio.mskcc.org/netbox.

### Conclusions

One of the central challenges confronting high-throughput cancer genomics is the ability to sift through the deluge of genomic profiling data and discern the true cancer signals from a general background of overall genomic instability. One of the most successful approaches to discerning the signal in mutation data or in copy number alteration data is to seek out those genes or regions which are altered at a frequency above a background random model. However, such an approach may only identify the most prominently altered genes, many of which may have already been known prior to the large investment in resequencing. And, while the focus on frequent regions of copy number alteration identifies many narrowly defined regions with few target genes, many regions contain dozens, if not hundreds of potential targets, and it is difficult to determine which of these represent the true targets.

In contrast to a purely frequency based approach to identifying cancer signals and candidate drivers, we and others [11] have proposed a network-based approach to the problem. Our approach is based on the hypothesis that tumors target specific biological modules of critical importance, and that such modules can be algorithmically identified based on network topology alone. Such an approach does, however, have certain drawbacks. First, network analysis is only as good as the network itself. Human interaction and pathway data remain sparse and fragmented, and we must assume that the Human Interaction Network (HIN) used here represents a small portion of the full human interactome [47]. Furthermore, interactions and pathways in our network are completely devoid of the context in which they were originally described, and we can only use the HIN as an approximate model for *in vivo* interactions. As a quality filter, we have also specifically chosen to include literature-curated interactions and pathways, but this may bias the network towards disease-associated genes. Second, distinguishing genes implicated by copy number alterations remains problematic, even when candidate genes are filtered through a network. For example, *KIT*, *KDR* and *PDGFRA* are all located at 4q12, a region of frequent amplification in GBM, and it is difficult to determine which one(s) are the true targets.

Despite these challenges, our network-based approach is able to automatically identify the main p53, Rb and PI3-kinase signaling modules, providing support for our hypothesis and our approach. It also identifies new candidate drivers, including *AGAP2*/*CENTG1*, a putative oncogene and an activator of the PI3K pathway, and three new modules of potential interest. Unlike the network-based approach proposed by Torkamani [11], our approach is based specifically on integrating mutation and copy number data, uses a high-quality literature curated network (as opposed to a network inferred from gene expression studies), and is not focused exclusively on rare mutations alone. We have also made our approach freely available within the NetBox software tool.

### Future Directions

We anticipate several possible future directions for our work. (1) More alteration data: the next phase of the TCGA GBM project will be sequencing over 6,000 gene and microRNA targets in several hundred additional GBM cases, as well as a number of tumor and paired normal complete genome sequences. This will provide much higher resolution, and a much broader coverage of genes, both of which will enable us to more effectively perform higher confidence network analysis and identify new candidate drivers and modules. (2) Larger network: we aim to broaden our network coverage by integrating the currently used literature curated networks with those derived from high-throughput protein-protein detection methods and microRNA prediction target programs, with appropriate quality filters. (3) Epigenetic data: we aim to integrate methylation, histone modification and unexpected expression changes into our analysis. (4) Quality of mutations: we plan to weight non-synonymous mutations with the likely functional impact based on analysis of residue conservation patterns and protein 3D structures. (5) Correlated events: correlation and anticorrelation between alteration events within and between modules can provide clues as to oncogenic history and as to likely vulnerabilities to targeted intervention. (6) Tumor subtypes: module alteration patterns, and possibly module structure is likely to differ between different tumor subtypes, such as the EGFR, PDFGRA and NF1 subtypes of GBM. (7) Diverse cancers: it will be of particular interest to investigate the common module features and characteristic differences for different cancer types, starting with the next two TCGA target cancers, ovarian and lung cancer. The extensions may provide additional insights into the nature of altered molecular processes in cancer.

## Methods

### GBM Mutation Data and Copy Number Analysis

Sequence mutation and DNA copy number alteration data for all TCGA GBM cases was obtained from the TCGA data portal [48]. Mutation data is only available for the 601 genes selected for resequencing in Phase 1 of the GBM project, and only for 91 GBM cases. For copy number data, 216 glioblastoma tumors were analyzed with RAE, as previously described [4], [49]. For all isoforms of autosomal genes in RefSeq (hg18), we discretized copy number using the multi-component model in RAE. We assign one of five putative aberration states to each isoform and tumor from overlapping regions of the unified breakpoint profile: (i) homozygous deletion (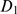 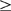
0.9), (ii) heterozygous loss (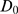 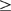
0.9 and 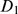 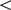
0.9), (iii) copy-neutral (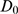 and 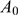 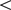
0.9), (iv) single-copy gain (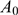 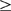
0.9 and 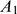 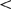
0.5), and (v) multi-copy amplification (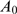 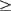
0.9 and 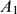 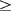
0.5). In the event of discontinuous coverage of a coding locus by regions that harbor intragenic breakpoints in copy-number segmentation, the region of extreme value in 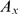 and 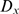 respectively determines the assigned state. Statistical significance of genomic alteration was assigned to genes as the minimum q-value from the one to many genomic regions that span each coding locus as determined by RAE. These include significance levels determined either by a model of total genomic gain/loss or a model designed to detect uncommon amplification and homozygous deletion events with little evidence of low-level changes (for details refer to [4], [49]).

An alteration frequency score for each gene was calculated, based on the 84 GBM cases with both sequence mutation and copy number data (hypermutators excluded). Each gene was considered altered if modified by a validated non-synonymous somatic nucleotide substitution, a homozygous deletion or a multi-copy amplification; all other copy number events were ignored, as was originally done in the original TCGA pathway analysis [4].

Network results were compared to the manually curated GBM network, as obtained from Figure S8 of the TCGA manuscript [4]. Individual gene and gene set alteration frequencies were obtained from the cBio Cancer Genomics Data Portal [50].

### Creation of the Human Interaction Network (HIN)

Interaction data from HPRD (Release 8: July 6, 2009) was obtained from the HPRD web site (http://www.hprd.org/) on Monday, September 28, 2009. Pathway data sets for Reactome (Release 29: June 24, 2009), NCI/Nature Pathway Interaction Database (November 12, 2008 Release), and the MSKCC Cancer Cell Map (May 19, 2006 Release) were downloaded in BioPAX format from Pathway Commons (http://www.pathwaycommons.org) on Monday, September 28, 2009.

**Figure 5:**
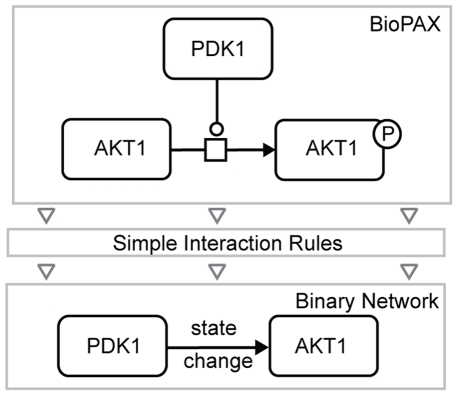
BioPAX to binary interaction mapping: example mapping rule. To integrate complex signaling pathway data into our Human Interaction Network (HIN), we have developed a set of rules for mapping subgraphs of biochemical networks to binary interactions. An example rule for mapping state changes, such as phosphorylation events, is shown.

Pathways in BioPAX are represented as sets of biochemical processes with inputs, outputs, and catalysts. A protein is often represented in multiple post-translational states, complexes, or cellular locations. However, most module discovery algorithms were developed for binary association networks where each biological entity is represented by a single node. To address this mismatch, we developed a set of rules for mapping subgraphs of biochemical networks to binary interactions. For example a phosphorylation reaction in BioPAX would be recognized as a “state change” interaction by the simple interaction rules (Figure 5). Similar reductions have been described by different groups in the past [51], [52] but these were limited to a single rule per study. Our current set of rules covers molecules participating in the same reaction or complex, and molecules catalyzing consecutive reactions, or co-controlling the same set of processes. The full list of rules and their explanations can be found at the Pathway Commons web site [53]. Rules were implemented in Java within a flexible and expandable framework and are available as a part of the open-source Paxtools library [54]. We have also made binary networks for each of the data sources in Pathway Commons available for download at: http://www.pathwaycommons.org.

All redundant edges were collapsed into single edges, and all self-directed edges were pruned from the network. After edge pruning, the final network consists of 9,264 genes and 68,111 interactions.

#### Identification of the GBM network and statistically significant linker genes

To determine the network of GBM altered genes, we began by creating an empty graph G. For each GBM altered gene X, we queried the HIN for all neighbors of X and placed these genes and interactions in G. Neighbor nodes of degree 1 are connected to exactly one altered gene, but do not inter-connect any altered genes, and are therefore immediately pruned from the network. Remaining neighbor nodes of degree ≥2 represent candidate linker genes, which connect two or more altered genes within the network. To identify statistically significant linker genes, we used the global degree of each linker gene within the HIN and the hypergeometric distribution to assess the probability that the linker gene would connect to the observed number of altered genes by chance alone. After FDR correction via Benjamini Hochberg [55], linker genes above a p-value threshold of 0.05 were pruned from the network. Finally, we queried the HIN for interactions between remaining linker genes, and added these to G.

### Module Detection

#### Modularity score

Modularity measures the fraction of edges in a network that connect nodes within-modules minus the expected value of the same quantity of edges in a network with the same module divisions but random connections between nodes [19]. It is measured by:
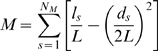
(1)


where 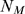 is the number of modules, 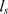 is the number of edges within module 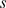, 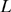 is the total number of edges in the network, and 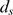 is the sum of the degrees of all nodes within module 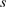 [19], [56]. If the number of within module edges is no better than random, the modularity value will approach 0; values approaching 1 indicate strong modular structure [19].

#### Module detection

We used the edge betweenness algorithm originally proposed by Girvan and Newman [18] to detect network modules, and implemented by the EdgeBetweennessClusterer in the Java JUNG library [57]. The JUNG implementation is a slight modification of the original algorithm and requires that one pre-specify the number of edges to be removed. As recommended in [19], we extended the JUNG implementation to sequentially remove all edges, and recalculate the network modularity after each edge removal, and automatically identify the number of edges which results in the optimal / maximum network modularity.

#### Global random gene set null model

To assess the level of global connectivity seen in the GBM network, we compared the size (number of nodes and edges) of the largest component in the network to the largest component generated by randomly selected sets of genes known to be present in the HIN. At each of 1000 iterations, we randomly selected 274 genes from the HIN and connected them via the original shortest path threshold and p-value cut-off parameters. We then determined the size of the largest component within this randomly generated network, and determined an empirical p-value by keeping track of the number of times this largest component equaled or exceeded the observed largest component.

#### Local random rewiring null model

To assess the statistical significance of the network modularity observed in the GBM network, we used a local rewiring algorithm, such that random networks maintain the same size and all genes maintain the same degree, but the choice of interaction partners is random [29]. For each random network, we calculate the network modularity, and calculate the average and standard deviation for the entire set of random networks. The observed modularity score is then converted into a z-score, or scaled modularity score to measure the deviation of the observed network from its random null model [30].

### Network Visualization and Module Analysis

Networks were visualized in Cytoscape [46]. Modules were visualized as discrete colors and gene alteration frequencies were visually resized in proportion to alteration frequency across the 84 analyzed GBM cases. Gene Ontology (GO) enrichment analysis was performed using DAVID [58], [59], using a background population of all genes in the Human Interaction Network (HIN). Correlation between discretized copy number calls and gene expression was assessed via ANOVA in R version 2.7.2.

### NetBox Software

The NetBox software, available at http://cbio.mskcc.org/netbox, is written in the Java and Python programming languages. It uses the Java HyperSQL embedded database to store the Human Interaction Network (HIN) and Entrez Gene information, and the Java JUNG library for all graph operations. To run the software, users must have Java 1.5 (or later) and Python 2.5 (or later) installed.
